# Identification of *WUSCHEL*-related homeobox (*WOX*) gene family members and determination of their expression profiles during somatic embryogenesis in *Phoebe bournei*

**DOI:** 10.48130/FR-2023-0005

**Published:** 2023-02-28

**Authors:** Miao Zhang, Xinyi Chen, Xiongzhen Lou, Yuting Zhang, Xiao Han, Qi Yang, Zaikang Tong, Junhong Zhang

**Affiliations:** State Key Laboratory of Subtropical Silviculture, School of Forestry & Bio-technology, Zhejiang A&F University, Lin'an, Hangzhou 311300, Zhejiang, P. R. China

**Keywords:** Phoebe bournei, Somatic embryo development, WOX, Hormones

## Abstract

*WUSCHEL*-related homeobox (*WOX*) transcription factor (TF)-encoding genes play crucial roles during embryo development. The function of *WOX* genes in embryonic development has been thoroughly studied in *Arabidopsis thaliana*, but little is known about their function in woody species, especially *Phoebe bournei*, an endemic and endangered species in China. In the present study, a total of 15 *WOX* genes were identified in* P. bournei*, and phylogenetic analysis resulted in their assignment to three typical clades: an ancient clade, an intermediate clade, and a modern/WUS clade. The gene structure and sequence characteristics and the physicochemical properties of WOX proteins were also analyzed. Promoter prediction indicated that *WOX* genes are likely involved in plant growth and development and hormone responses. Subsequently, we evaluated the expression patterns of *WOX* genes in response to auxin (IAA), abscisic acid (ABA), and methyl jasmonate (MeJA) treatments. According to tissue-specific expression patterns, we screened nine *WOX* genes that were present in embryonic calli and that might participate in the somatic embryogenesis (SE) of *P. bournei.* Furthermore, the expression profiles of these nine *WOX* genes during three phases of embryogenic calli development and three phases of somatic embryo development, namely, spheroid embryogenesis, immature cotyledon-producing embryogenesis and mature cotyledon-producing embryogenesis, were monitored. Overall, we systematically analyzed the expression patterns of *WOX* genes in *P. bournei* during SE, the information of which provides a basis for further elucidating the molecular mechanism through which *WOX* TFs function in *P. bournei* embryo development.

## INTRODUCTION

*Phoebe bournei*, a rare and endangered protected tree species that is unique to China, which produces excellent material and fragrance, can be used for the production of furniture and as an ornamental tree^[1]^ . However, few natural resources of this species are available, and this species undergoes a long juvenile phase. Currently, seed propagation is the main reproduction technique, and unstable yields driven by fruiting characteristics has a large impact on seedling production^[2]^.

Somatic embryogenesis (SE) is one of the most important techniques for tree breeding programs, but the mechanism underlying SE is poorly understood^[3]^. In angiosperms, a mature somatic embryo is induced from embryonic calli and subsequently develops into spherical, heart-shaped, torpedo, and cotyledon-producing embryos^[4]^. Moreover, regulation of the different stages of SE requires specific cell fate changes, and many transcription factors (TFs) are involved in this process. For example, *WUSCHEL* (*WUS*)*, WUSCHEL-related homeobox* (*WOX*),* BABY BOOM* (*BBM*),* AGAMOUS-like* (*AGL*),* LEAFY COTYLEDON* (*LEC*), *Receptor-Like Kinase* (*SERK*), and Vmyb Avian Myeloblastosis Viral Oncogene Homolog (*MYB*) genes function as indispensable regulators transforming nonembryogenic calli cells into embryogenic calli cells or driving changes between the different developmental stages of SE^[5−10]^. As such, SE requires precise transcriptional regulation. SE regeneration techniques have been determined for *P. bournei*^[11]^, and high-quality genomic data of this species have been released by our group^[12]^. However, the transcriptional regulatory mechanism behind the transitions among the different stages of SE in *P. bournei* remains elusive.

An increasing number of studies have shown that *WOX*s are extensively involved in plant organ regeneration, growth and development, stress responses, and other transcriptional regulatory processes, especially those that occur during SE^[13−17]^. In *Arabidopsis*, *AtWUS* was shown to be expressed at the proembryonic (16-cell) stage and is involved in subsequent maturation during SE^[18]^. *AtWOX2*, *AtWOX8*, and *AtWOX9* participate in polarity establishment during early embryonic development, and* AtWOX2* is expressed in apical cells, while *AtWOX8* and *AtWOX9* are expressed specifically in basal cells, which are indispensable for the correct establishment of the apical–basal axis^[19,20]^. Moreover, *PaWOX2* was also shown to be highly expressed in embryogenic cells in* Picea abies*^[21]^, and the overexpression of *PpWOX2* was shown to affect related traits of somatic embryos in* Pinus pinaster*^[17]^. In *Vitis vinifera*, *VvWOX2* and *VvWOX9* are expressed at high levels during SE and can be used as marker genes for SE^[22]^. Furthermore, *MtWOX9-1* was shown to increase the embryogenic capacity of recalcitrant plant species, e.g., *Medicago truncatula*^[23]^. These studies have shown that *WOX*s are crucial during the process of embryonic development or somatic embryo regeneration. Moreover, in woody plant species, global transcriptomic data and expression analysis have resulted in the identification of *WOXs* expressed during SE in *Dimocarpus longan*, hybrid sweetgum, and *Elaeis guineensis*, suggesting that *WOXs* are functionally conserved in woody plants species^[7,9,24]^. Based on this, understanding the dynamic relationship between *WOX*s and SE in *P. bournei* is helpful for optimizing the somatic embryo regeneration system and creating a large number of clones rapidly.

Previous studies have shown that overexpression or ectopic expression of embryogenesis-related TFs can induce the SE process. Another way is to apply exogenous phytohormones^[25]^. Adding exogenous phytohormones to media can affect the morphology and quality of SE in many species^[26]^. The interactions between phytohormones and TFs has been under increasing scrutiny. Several studies have shown that some TFs, such as *LEC2*, *BBM*, and *WUS*, are regulated by auxin synthesis, transport, and responses during SE^[10,27,28]^. Correct establishment of the auxin gradient and PIN1-mediated auxin transport were shown to affect the expression level of *WUS*, which in turn affected the status of the embryonic calli^[27]^. *LEC2* and *BBM* transcriptionally regulate the endogenous auxin (IAA) biosynthesis-related genes *YUCs/TAA*s and increase the DR5 auxin response, further maintaining somatic embryo growth^[10,28,29]^. Furthermore, *LEC2* was shown to bind directly to the early embryonic marker genes *WOX2* and *WOX3,* triggering SE^[5,25]^. Abscisic acid (ABA) is another important hormone involved in SE, especially during embryo maturation. Application of exogenous ABA to the media was shown to induce embryo maturation and prevent early germination in *Carica papaya*, *Pseudotsuga menziesii*, and *Phoenix dactylifera*^[30−32]^. Methyl jasmonate (MeJA) plays a function similar to that of ABA in promoting mature SE. MeJA functions synergistically with ABA, but the effects of MeJA cannot replace the effects of ABA^[33]^. In *Liriodendron* hybrids, MeJA was shown to increase both SE and the maturation rate and decreased the deformation rate^[34]^. However, studies on the relationships between MeJA and *WOXs* are lacking. Taken together, these results suggested that TFs and hormones jointly regulate plant SE. In *P. bournei*, how *WOXs* respond to hormones during SE has not been thoroughly characterized. So we preliminarily explored the expression patterns of *WOX* under auxin, ABA, and MeJA treatments.

In the present study, 15 *WOX* genes were identified across the *P. bournei* genome, and their gene structures and protein sequences were characterized. Then, the expression patterns of *WOXs* among six tissues and at different stages of SE were determined. To elucidate how these *WOXs* respond to hormones, their expression levels in response to auxin, ABA, and MeJA were analyzed. Our results revealed *WOX* members in *P. bournei* and several possible associations between *WOXs* and plant hormones. The results of this study will provide further insight into the function of *WOXs* involved in regulating SE in woody plant.

## MATERIALS AND METHODS

### Plant materials and growth conditions

The half-sibling family of* P. bournei* designated 'WY1' was cultivated in the greenhouse. The epicotyls, stem tips, roots, stems, and leaves of three-month-old seedlings and embryogenic calli induced from immature embryos were frozen in liquid N_2_ and used for semiquantitative analysis of *PbWOX* genes. Growth of embryonic calli was induced in cotyledon-stage embryos of the 'WY1' mother tree and subcultured at 24 °C in the dark, as described in our previous study^[11]^. Somatic embryos of* P. bournei* at six developmental stages, including three stages of calli, the globular embryo stage, and immature and mature cotyledon-producing embryo stages, were collected under a stereomicroscope (OLYMPUS, Beijing, China) and then frozen in liquid N_2_ for RNA extraction. With respect to calli growth in liquid media for hormone treatment, 0.1 g of calli was transferred to liquid media supplemented with 100 μM IAA, ABA, and MeJA for 3, 6, 12, 24, and 48 h. Calli in untreated liquid media were used as controls. Sampling was performed at the same time, and three replicates were included.

### Identification and phylogenetic analysis of *PbWOXs*

The PbWOXs were identified by two methods. Firstly, using the hidden Markov model, we downloaded the sequence of the conserved homeobox domain of the WOX (PFAM00046) from an online website (http://pfam.xfam.org), and the hmm search module in HMMER (version 3.1) software was used to search the protein sequences of the *P. bournei* genome^[12]^. The threshold was set to < E^−^^20^. Secondly, we downloaded 15 AtWOXs proteins sequence from the TAIR database (www.arabidopsis.org), then used them as query sequences to perform the BLASTp search (E-value < 1e-5) with *P. bournei* protein sequences. By combining the two methods, candidate sequences without a homeobox domain were omitted. The 15 obtained PbWOX protein sequences were subjected to MUSCLE alignment of MEGA (version 7.0) together with the sequences of 15 AtWOXs, 13 OsWOXs, 18 PtWOXs, eight AtriWOXs, seven SmWOXs, three PpaWOXs, one OstuaWOX, and one OstluWOX protein downloaded from the online Plant Transcription Factor Database (PlantTFDB) website (http://planttfdb.cbi.pku.edu.cn). The neighbor-joining (NJ) method with 1000 bootstrap repetitions was subsequently used to construct a phylogenetic tree, and the other parameters were set to their default.

### Characterization of *PbWOX* genes and proteins

Exons and introns of individual *PbWOXs* were visualized *via* the online software Gene Structure Display Server (GSDS) (version 2.0) (http://gsds.gao-lab.org), and Multiple Em for Motif Elicitation (MEME) (version 5.11) (http://meme-suite.org/) was used to predict the motifs of the PbWOX family proteins. The ProtParam (https://web.expasy.org/protparam/) online website was subsequently used to predict the physicochemical properties of PbWOX family members, such as their number of amino acids, molecular weight, and isoelectric point. ClustalX (version 1.81) was used for multiple sequence alignment to confirm the presence of WUS-box domain and the homeobox domain. The genome sequence and gene annotation information file was added to the TBtools GFF3 Sequence Extractor submenu, the upstream bases was set to 2000, the upstream CDS 2.0 kb of all genes in *P. bournei* were obtained. Then 2.0 kb upstream promoter sequences of 15 *PbWOX* genes were obtained from the TBtools quick fasta extractor submenu^[12,35]^. Finally, we uploaded the obtained file to an online site PlantCARE (http://bioinformatics.psb.ugent.be/webtools/plantcare/html/) to analyze *cis-*acting elements.

### RNA extraction and gene expression analysis

Total RNA was extracted using a RNAprep Pure Plant Kit (TIANGEN, Beijing, China). Then, the RNA was quantified by a Nanodrop ND-1000 spectrophotometer and checked according to the A260/280 nm and A260/A230 nm values. Subsequently, cDNA was synthesized using a PrimeScript^TM^ RT Reagent Kit with gDNA Eraser (Perfect Real Time) (Takara, Dalian, China), and each RNA sample was 2000 ng. The obtained cDNA was subsequently diluted five times for quantitative RT‒PCR.

Specific primers of the 15 *PbWOX* genes were designed using the Primer 3 online website (http://bioinfo.ut.ee/primer3-0.4.0/), and the sequences of these primers are listed in Supplemental Table S1. The expression levels of the *PbWOX* genes were detected *via* quantitative RT–PCR and a CFX 96-well Real-Time PCR System (Bio-Rad, USA). The qPCR mixture volume was 10 μL, which comprised 5 μL of 2× ChamQ^TM^ SYBR qPCR Master Mix, 0.4 μL of cDNA, 0.2 μL of forward primer, 0.2 μL of reverse primer, and 4.2 μL of ddH_2_O. The PCR was carried out as follows: predegeneration at 95 °C for 1 min, 45 cycles of denaturation at 95 °C for 10 s followed by annealing at 57 °C for 10 s, and extension at 72 °C for 20 s. *PbEF1α* was used as an internal control, and the relative gene expression levels were calculated according to the 2^−ΔΔCᴛ^ method^[36]^.

**Table 1:** Subclass information of *WOX*s among *P. bournei* and other representative species.

Taxonomic group	Species	Ancient clade	Intermediate clade	Modern/ WUS clade	Total
Dicots	*A. thaliana*	3	4	8	15
	*P. trichocarpa*	6	7	11	18
Monocots	*O. sativa*	1	6	6	13
Magnoliales	*P. bournei*	3	4	8	15
Amborellales	*A. trichopoda*	1	2	5	8
Pteridophyta	*S. moellendorffii*	6	1	−	7
Bryophyta	*P. patens*	3	−	−	3
Chlorophyta	*O. tauri*	1	−	−	1
	*O. lucimarinus*	1	−	−	1

### Statistical analysis

All the treatments were performed at least three times. The data were subjected to ANOVA and Duncan's multiple range test at the 5% significance level *via* SPSS (version 26.0) software.

## RESULTS

### Identification and phylogenetic analysis of PbWOXs

**Figure 1:**
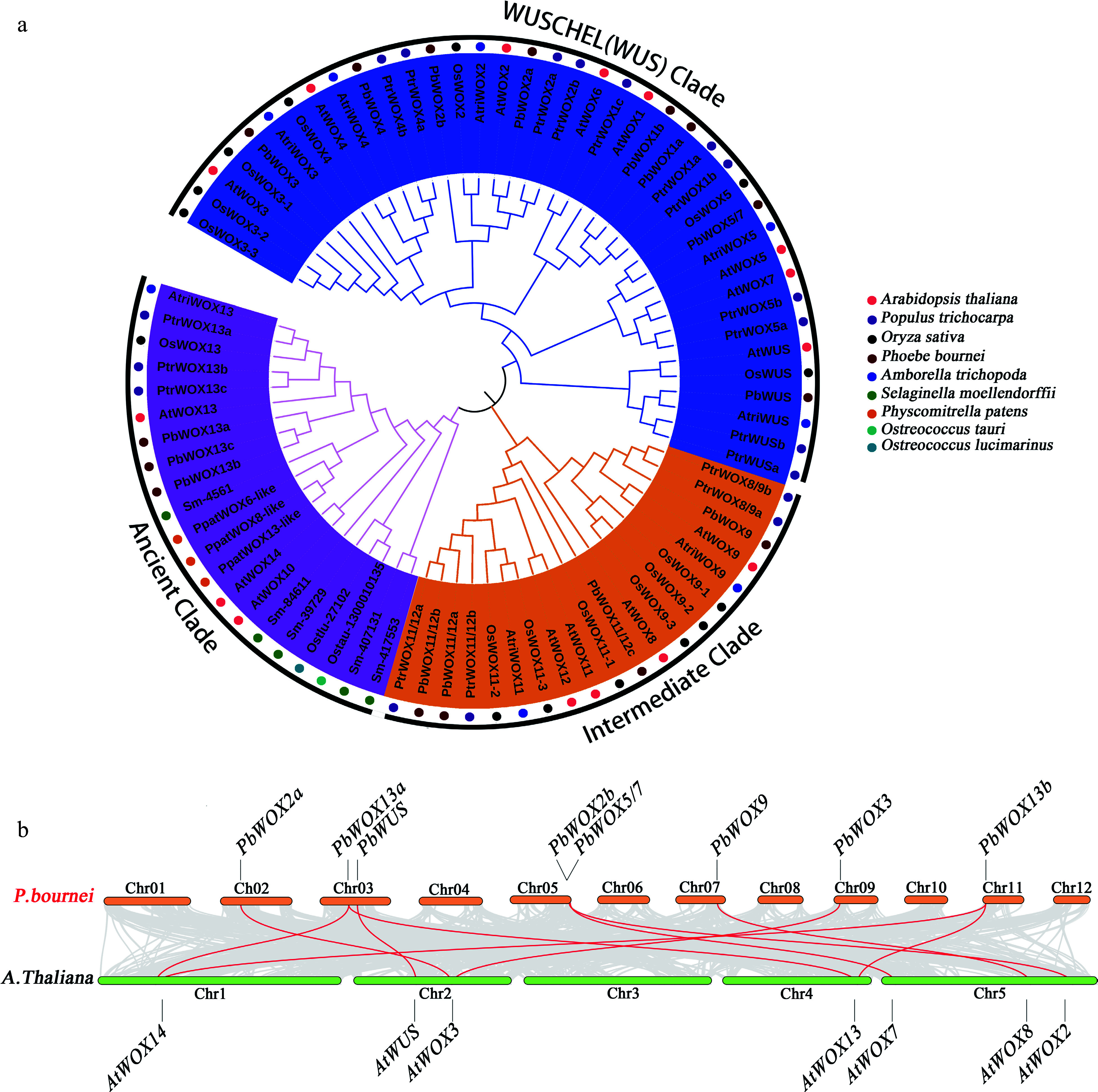
Phylogenetic relationships of PbWOX proteins. (a) NJ tree constructed of the amino acid sequence of WOXs from* Phoebe bournei* (Pb)*, Arabidopsis thaliana* (At)*, Populus trichocarpa* (Pt),* Oryza sativa* (Os)*, Amborella trichopoda* (Atri)*, Selaginella moellendorffii* (Sm)*, Physcomitrella patens* (Ppa)*, Ostreococcus tauri* (Ostau)* and Ostreococcus lucimarinus* (Ostlu)*.* (b) Synteny analysis of *WOX* genes between *P. bournei* and *A. thaliana*. Gray lines indicate all synteny blocks in the genome, and the red lines indicate duplicated *WOX* gene pairs.

After performing hidden Markov model (HMM) searches and removing redundant and/or sequences without the homeobox domain, we identified 15 PbWOX members. Phylogenetic analysis of 15 AtWOXs, 18 PtWOXs, 13 OsWOXs, eight AtriWOXs, seven SmWOXs, three PpaWOXs and WOX protein sequences from two green algal species resulted in the assignment of the 15 PbWOX genes to an ancient branch, an intermediate branch and a modern/WUS branch (Table 1). Specifically, the ancient branch consisted of three PbWOXs (PbWOX13a, PbWOX13b, and PbWOX13c); the intermediate branch consisted of four PbWOXs, namely, PbWOX9, PbWOX11/12a, PbWOX11/12b, and PbWOX11/12c, which were classified into two subclasses; and the remaining eight members, namely PbWUS, PbWOX1a, PbWOX1b, PbWOX2a, PbWOX2b, PbWOX3, PbWOX4 and PbWOX5/7, were assigned to the modern/WUS branch (Fig. 1a).

However, the number of *PbWOX* genes was the same as that of *Arabidopsis* (Fig. 1a). Nonetheless, *PbWOXs* probably expanded differently than did those of *Arabidopsis*. For example, three homologs of *AtWOX11/12* and *AtWOX13*, two homologs of *AtWOX1* and *AtWOX2*, one homolog each of *AtWUS*, *AtWOX3*, *AtWOX4*, *AtWOX5/7*, and *AtWOX9*, and no homologs of *AtWOX6* and* AtWOX10* were found in* P. bournei* (Fig. 1b).

### Physicochemical properties and analysis of conserved motifs of PbWOXs

**Figure 2:**
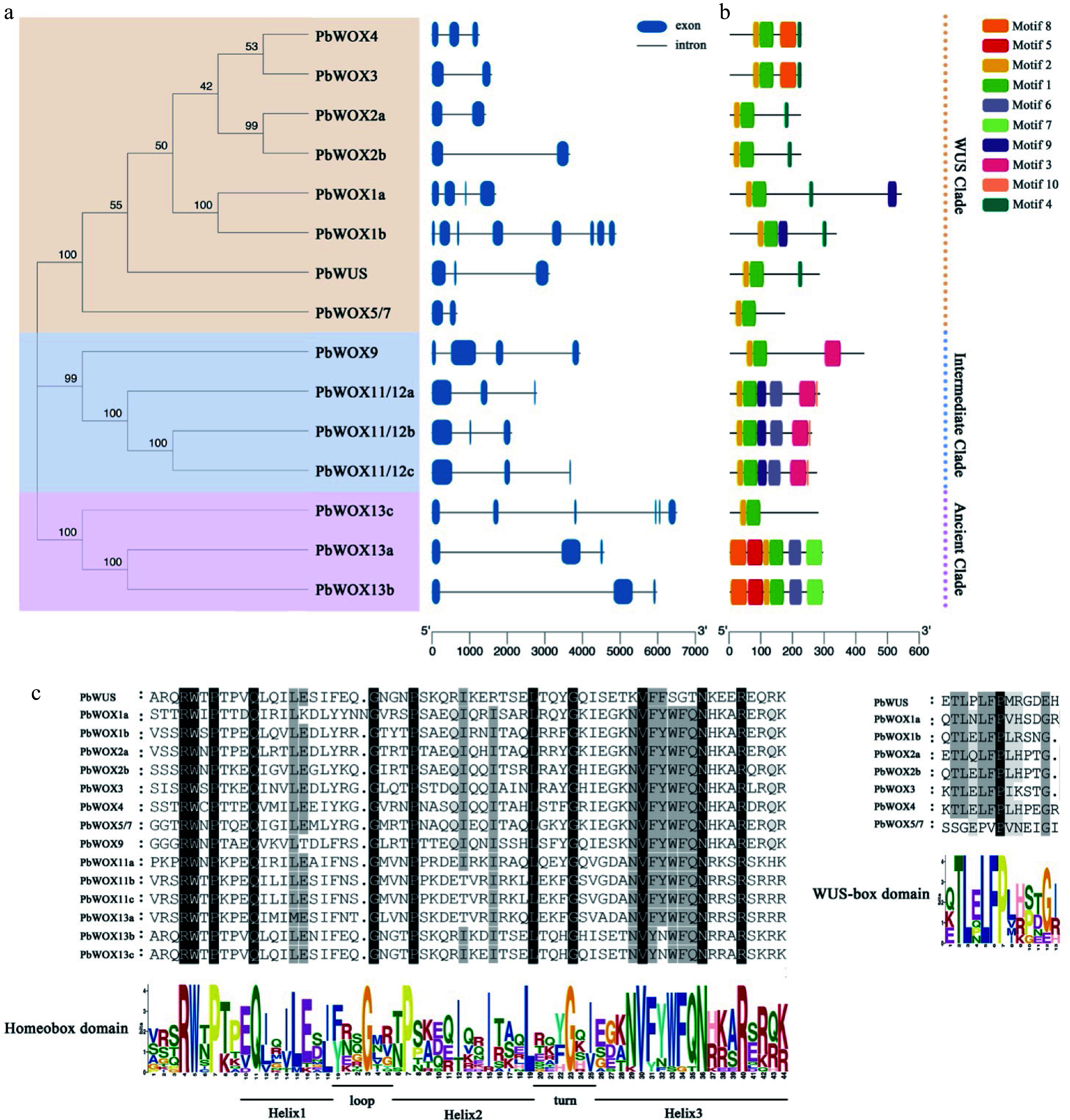
Information on the PbWOX genes and proteins. (a) Phylogenetic tree and gene structure. (b) Architecture of conserved protein motifs. (c) Multiple sequence alignment.

A sequence analysis of the PbWOXs showed that PbWOX1b comprised the largest number of amino acid residues (528) and had the largest molecular weight (59.19 kD). Conversely, PbWOX5/7 comprised 169 amino acid residues and had the smallest molecular weight (19.37 kD). All *PbWOX* genes contain introns, the number of which ranged from two to eight (Fig. 2a). Then, to better understand each member of the PbWOXs*,* we predicted the physicochemical properties by the use of an online website. The theoretical isoelectric point of PbWOX was found to be between 5.48 (PbWOX11/12c) and 9.93 (PbWOX13c) (Table 2).

**Table 2:** Summary of the *PbWOX* gene family members.

Gene ID	Gene name	Orthologous in Arabidopsis	Theoretical pI	Molecular weight	Number of amino acids
OF24054-RA	*PbWUS*	*AtWUS*	8.58	31622.96	276
OF03970-RA	*PbWOX1a*	*AtWOX1*	9.37	37209.6	328
OF11837-RA	*PbWOX1b*	*AtWOX1*	8.89	59188.97	528
OF19048-RA	*PbWOX2a*	*AtWOX2*	7.09	24524.5	218
OF05256-RA	*PbWOX2b*	*AtWOX2*	6.83	24496.61	219
OF16243-RA	*PbWOX3*	*AtWOX3*	9.05	22752.66	194
OF04424-RA	*PbWOX4*	*AtWOX4*	8.25	24797.85	220
OF05362-RA	*PbWOX5/7*	*AtWOX5, AtWOX7*	9.51	19371.68	169
OF24594-RA	*PbWOX9*	*AtWOX9*	7.19	45272.57	413
OF22069-RA	*PbWOX11/12a*	*AtWOX11, AtWOX12*	5.68	30055.89	268
OF11766-RA	*PbWOX11/12b*	*AtWOX11, AtWOX12*	5.95	30330.35	277
OF28194-RA	*PbWOX11/12c*	*AtWOX11, AtWOX12*	5.48	27450.91	252
OF25757-RA	*PbWOX13a*	*AtWOX13*	5.91	32705.61	288
OF14063-RA	*PbWOX13b*	*AtWOX13*	6.10	32380.27	286
OF07768-RA	*PbWOX13c*	*AtWOX13*	9.93	31294.66	272

Motif 1 and motif 2 were detected in all 15 PbWOXs, motif 3 was specific to the members of the intermediate clade, motif 4 (T-L-X-L-F-P-X-X, where X indicates any amino acid) was present in all members of the modern/WUS clade except PbWOX5/7, and motif 5 was specific to PbWOX13a and PbWOX13b (Fig. 2b). There are residues composing homeobox domain motifs that contain three helixes spaced by one loop and one turn (Fig. 2c). Eight members in the modern/WUS clade shared a WUS-box domain (Fig. 2c).

### Prediction of *cis-*acting elements in the *PbWOX* promoters

**Figure 3:**
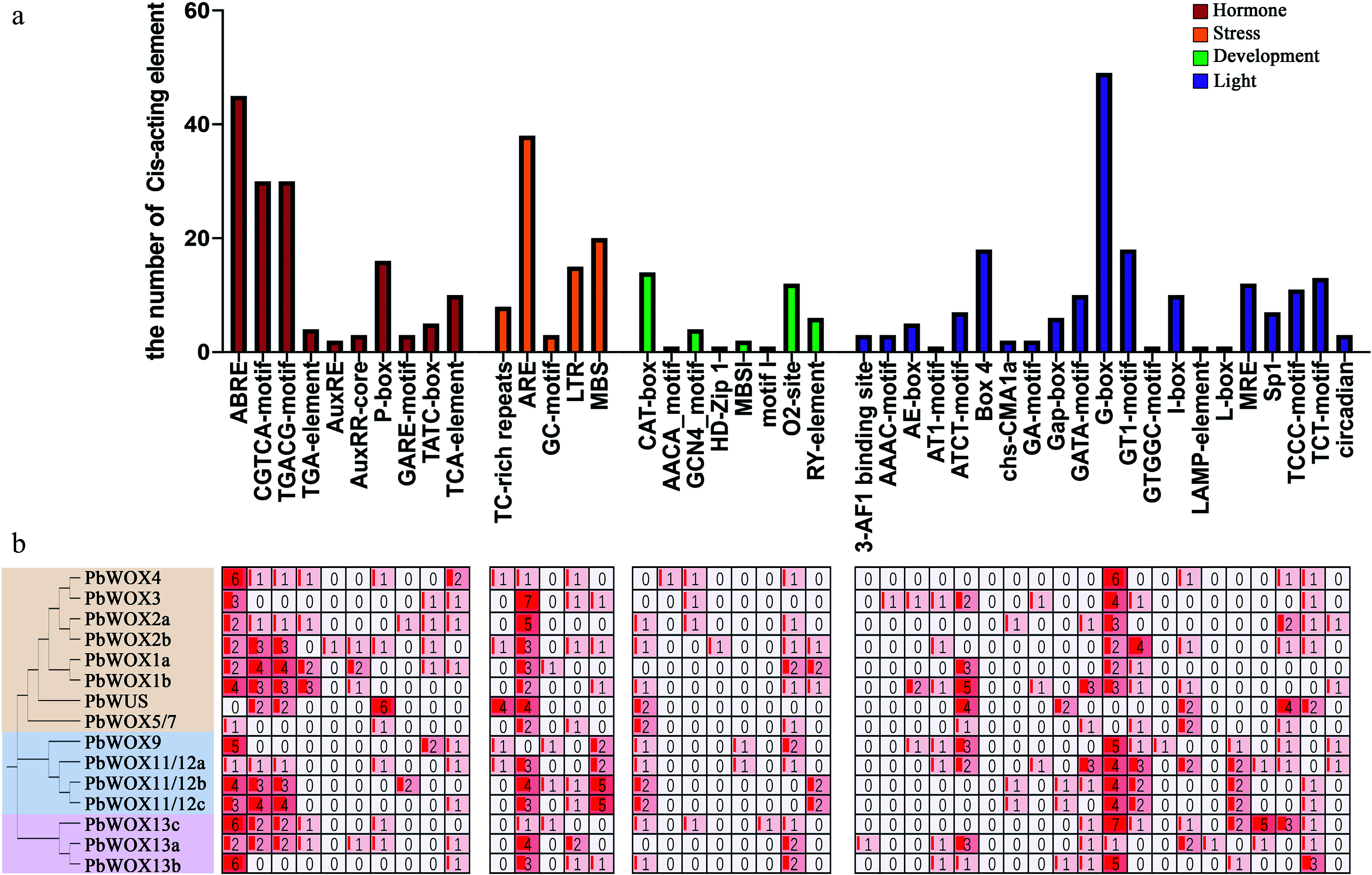
Predicted *cis*-acting elements in *PbWOX* promoters. (a) Frequency of *cis-*acting elements in the 2.0 kb upstream regions of *PbWOXs*. The corresponding colored bar chart indicates the occurrence of different *cis-*acting elements. (b) Number of *cis-*acting elements in each *WOX* gene.

The *cis-*acting elements in the promoter region of *PbWOXs* were divided into four main categories: light-related, hormone-related, stress-related and development-related. (Fig. 3). Specifically, the light response elements constituted the largest proportion, of which the number of G-box elements was the largest. Several other elements involved circadian rhythm were also detected. The hormone-responsive elements included 45 ABA-responsive elements (ABREs), 30 MeJA-responsive elements (CGTCA motif–containing elements), 24 gibberellin (GA)-responsive elements (P-boxes, GARE motif–containing elements, TATC-boxes), 10 salicylic acid-responsive elements (TCA-elements), and nine auxin-responsive elements (TGA-elements, AuxREs, AuxRR-core elements). Abiotic stress response elements were predicted with 38 regulatory anaerobic inductor elements (ARE), 20 drought-responsive elements that could bind MYBs (MBSs), 15 low-temperature–responsive elements (LTRs), eight defense- and stress-responsive elements and five anoxic-specific induction-responsive elements. Moreover, in development-related *cis-*acting elements, 14 CAT boxes, 12 O2-sites, and six RY elements were predicted, respectively. In the *PbWOX* promoters, the most common *cis*-acting elements were G-boxes (light-related), ABREs (ABA-related), CGTCA motif-containing elements (MeJA-related) and AREs (drought-related). This result implied that *PbWOX* participated in plant growth process and stress response.

### Diversified expression patterns of* PbWOXs* among tissues

**Figure 4:**
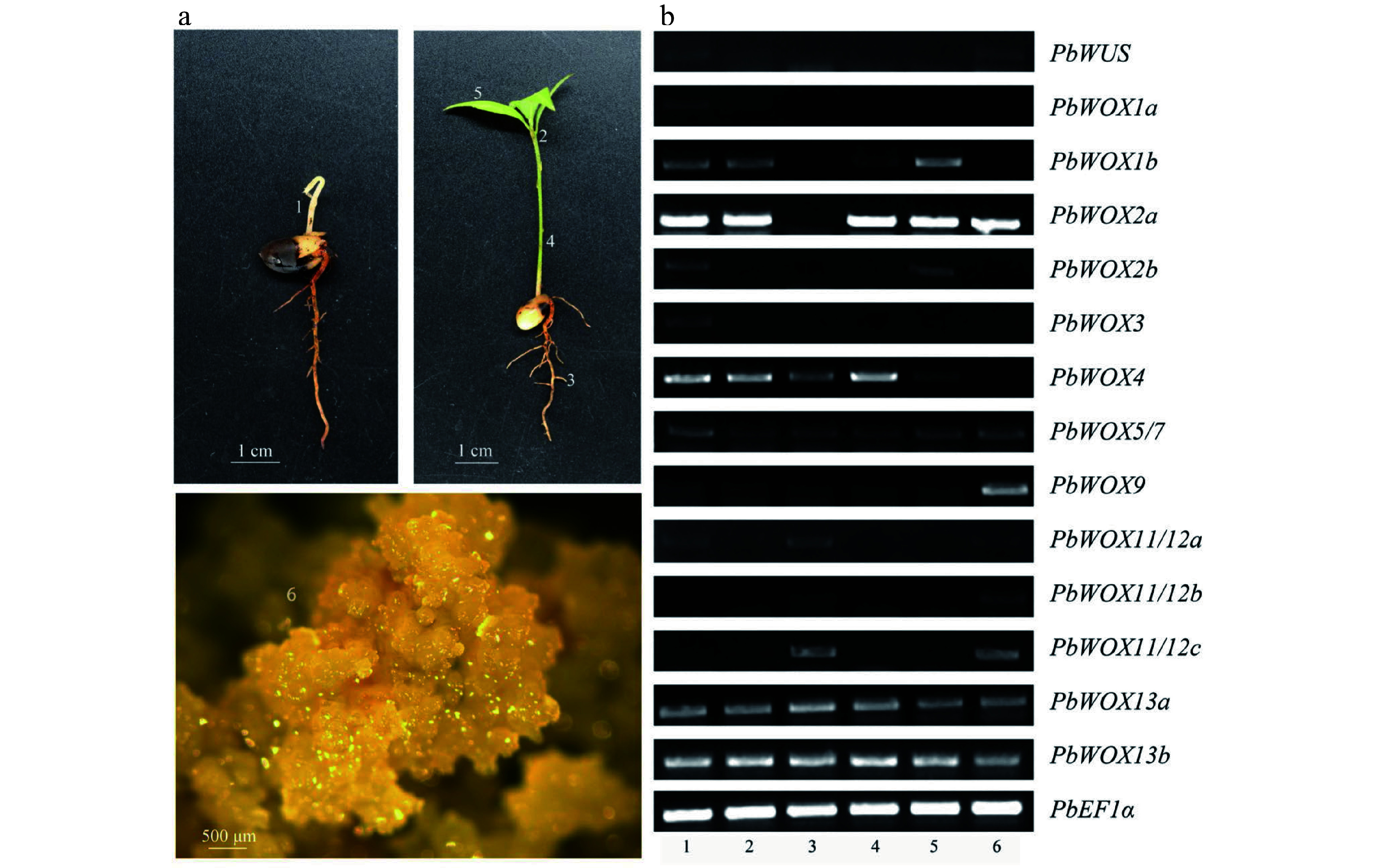
Semiquantitative analysis of *PbWOXs* in different tissues. (a) Tissue samples, 1 - epicotyl, 2 - stem tip, 3 - root, 4 - stem, 5 - leaf, 6 - calli. (b) Semiquantitative PCR electropherogram.

To further understand the potential roles of *PbWOXs* during different developmental stages and at different physiological status, semi-qPCR was used to study the expression patterns of 15 *PbWOXs* in six tissues. The expression levels of *PbWOXs* varied significantly among the tissues (Fig. 4). Specifically, five genes, namely, *PbWOX2a*, *PbWOX5/7*, *PbWOX9*, *PbWOX13a*, and *PbWOX13b*, were expressed in almost all the tissues, while *PbWUS*, *PbWOX1a*, *PbWOX2b* and *PbWOX3* were highly expressed in the epicotyls, with low or no expression in the other tissues. In addition, *PbWOX11*/*12a*, *PbWOX11/12b* and* PbWOX11/12c* were highly expressed specifically in both the roots and embryogenic calli, while expression of *PbWOX1b* and *PbWOX4* was nearly absent in the calli. In total, nine *PbWOXs* were expressed in embryogenic calli, and thus, these genes may be involved in the SE of *P. bournei*; *PbWOX2a* exhibited the highest expression level.

### Expression patterns of *PbWOXs* during SE of *P. bournei*

**Figure 5:**
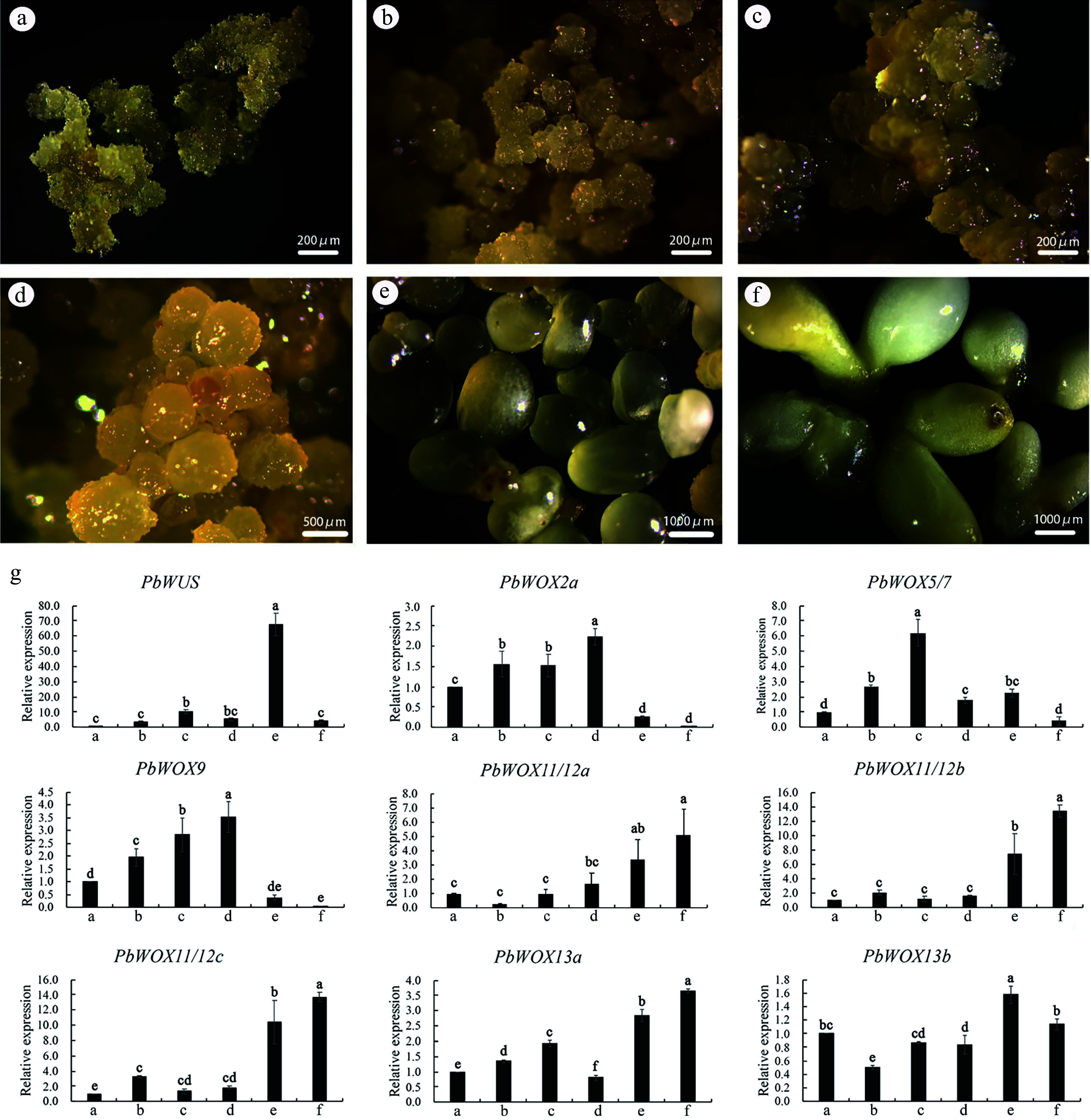
Expression patterns of *PbWOXs* during SE of *P. bournei*. (a) Calli-1. (b) Calli-2. (c) Calli-3. (d) Globular embryo. (e) Immature cotyledon-producing embryo. (f) Mature cotyledon-producing embryo. (g) Analysis of gene expression *via* qPCR. The data are the means ± SDs of three biological replicates. The values followed by the same letter are not different according to Duncan’s multiple-range test. *PbEF1α* was used as an endogenous control.

Previous studies have shown that *WOXs* play important roles during SE. The expression levels of nine *PbWOXs* were analyzed in calli at three different developmental stages (Fig. 5a−c) and in embryos at three different developmental stages (Fig. 5d−g). Embryonic calli were induced by immature zygotic embryos (Fig. 5a); then, the embryonic calli developed to the second stage (Fig. 5b) after two or three rounds of propagation, and the calli developed to the third stage (Fig. 5c) after two rounds of propagation. Globular embryos (Fig. 5d), immature cotyledon-producing embryos (Fig. 5e) and mature cotyledon-producing embryos (Fig. 5f) were also selected. The qPCR results showed that the expression levels of *PbWOX2a* and *PbWOX9* increased during embryogenic calli development but decreased as the embryos matured. *PbWUS* was specifically and highly expressed in the immature cotyledon-producing embryos. The expression level of *PbWOX5/7* increased during calli development but decreased after calli differentiation. Three homologous genes, *PbWOX11/12* and *PbWOX13a*, were highly expressed in cotyledon-producing embryos, and their expression peaked upon maturity (Fig. 5g).

### Expression profiles of *PbWOXs* in response to hormone treatment

**Figure 6:**
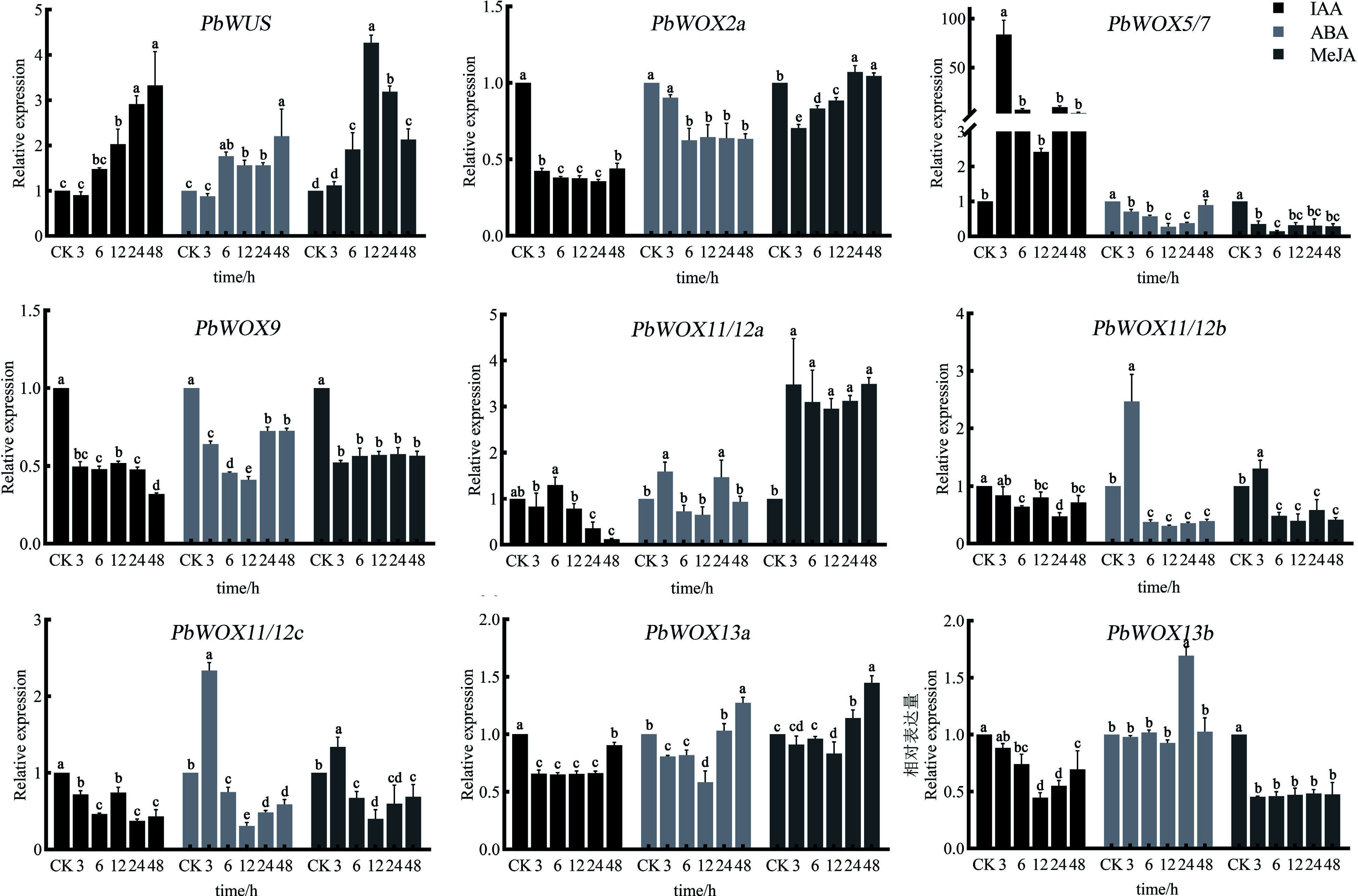
Relative expression levels of *PbWOXs* under hormone treatment. The data are the means ± SDs of three biological replicates. The values followed by the same letter are not different according to Duncan's multiple-range test. *PbEF1α* was used as an endogenous control.

With respect to the *cis-*acting elements of *PbWOXs*, we investigated the expression patterns of *PbWOXs* in response to auxin, ABA, and MeJA (Fig. 6). Under IAA treatment, *PbWUS* expression was induced and increased continuously as the treatment duration increased; *PbWOX5/7* was strongly induced after 3 h of treatment, after which the expression level decreased. The expression levels of *PbWOX2a* and *PbWOX9* significantly decreased, and the expression levels of *PbWOX11/12b*, *PbWOX11/12c*, *PbWOX13a*, and *PbWOX13b* also slightly decreased.

*PbWUS* was also induced in response to ABA treatment, while *PbWOX2a*, *PbWOX9*, and *PbWOX13b* were inhibited. The expression levels of *PbWOX5/7* and *PbWOX13a* decreased, reached their lowest level after 12 h of ABA treatment, and then gradually increased. *PbWOX11/12b* and *PbWOX11/12c* showed similar expression patterns; their expression increased after 3 h but then decreased. *PbWUS* expression was induced in response to MeJA treatment, peaked at 12 h, and then gradually decreased. *PbWOX5/7*, *PbWOX9* and *PbWOX13b* expression was inhibited significantly. *PbWOX11/12b* and *PbWOX11/12c* expression increased after 3 h but then decreased.

## DISCUSSION

### Characteristics and expression of the *WOX* family members in *P. bournei*

*WOXs* are specific to plants and largely involved in key developmental processes, especially those associated with somatic cell regeneration. With the publication of many plant genome sequences, *WOX* genes have been identified in several plant species. In the present study, we identified 15 *PbWOXs*, same as the number in *Arabidopsis*^[37]^, and different orthologous revealed that chromosomal duplication events may occur in *P. bournei.* Furthermore, the length of introns showed regular characteristics across different clades. For instance, genes in the intermediate clade contained shorter intron sequences than did those of ancient clade, and five genes in the modern/WUS clade had the shortest intron. Taken together, these results suggested that the intron fragments underwent refinement during the evolution of the *PbWOX* genes. A similar phenomenon was observed in *Camellia sinensis*^[38]^, which was exemplified by most *WOX* introns in members of the modern/WUS clade are much shorter than those in the ancient clade. In addition, compared with that in algae, ferns and other more ancestral plant species with one or two members, the* WOX* family gene in woody plant species has expanded in number and evolved in terms of sequence.

Tissue-specific expression of a gene implies that the gene plays an indispensable role in certain tissues. We found that, like those in *Arabidopsis*, the *WUS* genes in* P. bournei* were mainly expressed in the epicotyls and shoot apical meristems (SAMs), but this is unlike the patterns of other popular genes, which are expressed in the SAMs, roots, stems, and leaves^[39]^. These results suggested that *PbWUS* might play a crucial role in maintaining the differentiation of the SAM. In *Arabidopsis*, *AtWOX4* participates in TDIF-TDR-WOX4 signaling to maintain vascular meristem organization during secondary growth^[40]^, which is similar to what occurs in poplar^[41,42]^. Here, *PbWOX4* was also highly expressed in the stems; thus, this gene may have a function in *P. bournei* like that of its homologs in *Arabidopsis* and poplar. In addition, *PbWOX4* was also expressed in the roots, leaves, and other plant tissues except embryogenic calli, suggesting that this gene is not involved in plant regeneration or development *in vitro*. Like *AtWOX11*, *PtoWOX11/12a*, and *PtoWOX11/12b*, three members, namely, *PbWOX11/12a*, *PbWOX11/12b*, and *PbWOX11/12c,* were expressed in the roots and embryogenic calli. Previous studies have shown that *WOX11* is involved in adventitious root formation, which has an essential function in root regeneration during *de novo* plant regeneration^[43−46]^. Therefore, it was speculated that these three *PbWOX11* members might participate in calli propagation and/or root regeneration in* P. bournei*.

### Expression patterns reveal that *PbWOX* participates in SE

SE is one of the important mechanisms of plant asexual reproduction and is subject to complex transcriptional regulation, which in turn enables precise cell fate transitions and the formation of a complete plant. This hierarchical transcriptional regulatory network structure for SE has been revealed in *Arabidopsis*; in this process, *WOX2* and *WOX3* are the key TFs that induce SE^[5]^. According to the tissue expression patterns among tissues, we identified nine *WOX* genes that were expressed in embryogenic calli—the early stage of SE.

*WUS* plays a crucial role in embryogenesis by promoting the fate of cells to transform and develop into embryos, and *WUS* can also drive the activity of embryonic stem cells. An earlier study showed that *WUS* is expressed in the four inner apical cells of 16-cell embryos and promotes the formation of the SAM during embryo development, and overexpression of *WUS* promotes the formation of high-frequency SE. Like in other species, such as *Coffea canephora*^[47]^,* Medicago truncatula*^[48]^, and *Gossypium hirsutum*^[15]^, *WUS* overexpression resulted in an increased SE induction ratio. In our study, the expression level of *WUS* significantly increased in the late stage of embryogenic calli and increased significantly again at the immature cotyledon-producing embryo stage. These results indicated that *PbWUS* promotes the proliferation of embryogenic calli, affects the establishment of cell axial polarity, such as the formation of apical bud meristems during embryonic development in plants, and especially promotes the transition to cotyledon-producing embryos.

In addition to *WUS*, *WOX2* and *WOX9* are the most reported *WOX* genes involved in plant SE. In the present study, *PbWOX2a* and *PbWOX9* exhibited similar expression patterns, which were exemplified by higher expression levels observed at the embryogenic cell stage and during early somatic embryo formation. In *Arabidopsis*, it has been proposed that *AtWOX2* and *AtWOX9* play crucial roles in apical–basal axis formation during embryo development^[19]^. *AtWOX2* is expressed in the apical cell, whereas *AtWOX9* is expressed in the basal cell. These genes expressed at specific sites drive the fate of cells in the embryo. In grapevine, both *VvWOX2* and *VvWOX9* are labeled marker genes of early embryogenic phases^[22]^. In addition, *WOX2* and *WOX9* were found to play crucial roles in the early stage of SE in the gymnosperm *Picea abies*^[21,49]^. Therefore, *PbWOX2a* and *PbWOX9* might be marker genes for early embryonic development of *P. bournei*.

*WOX11* has been reported to be an important upstream gene involved in the generation of root system architecture and to promote adventitious root formation during *de novo* root organogenesis from leaf explants^[44,50]^, but this gene has not been found to be related to root regeneration in plant SE. We noted that *PbWOX11/12a*, *PbWOX11/12b*, and *PbWOX11/12c* were all detected in the embryogenic calli, specifically in immature and mature cotyledon-producing embryos. A similar phenomenon has been observed in grapevine, exemplified by* VvWOX11* being highly expressed in torpedo-stage and cotyledon-producing embryos^[22]^. These findings further support that *WOX11/12* might play an important role in the later stage of somatic embryo development and is probably related to root development, but whether *WOX11/12* is involved in root primordium formation remains to be confirmed.

In *P. bournei*, two *WOX13* genes orthologous to *AtWOX13* were detected in calli and somatic embryos, but their expression patterns differed. The expression level of *PbWOX13a* gradually increased with embryo development, while that of *PbWOX13b* showed no significant change. To our knowledge, *WOX13* is expressed ubiquitously and participates in calli formation and organ reconnection in *Arabidopsis*^[51]^. However, the molecular regulatory roles of *WOX13* during somatic embryo regeneration remain unclear, and expression profiles have been reported in only *Vitis vinifera*, in which three *VvWOX13* genes exhibited low expression levels in somatic embryos, and the expression profile was unaffected by environmental changes^[22]^. Our data showed that two *PbWOX13s* also exhibited ubiquitous expression patterns. Nevertheless, *PbWOX13b* expression seemingly changed nonsignificantly during embryonic calli induction and mature cotyledon-producing embryos, while the expression level of *PbWOX13a* slightly increased in the later stage of somatic embryo development. Taken together, these results suggested that *PbWOX13a* might play a regulatory role at the later stage of somatic embryo development.

### Response of *PbWOX* genes to various hormones during SE

SE is a highly efficient method for plant regeneration^[52]^. Overexpression of *WUS,* *WOX2*, *WOX9*, *BBM*, and *SERK* is an efficient way to induce SE, and application of plant growth regulators such as auxin, MeJA, ABA, and GA is another useful method^[6,47,53−55]^. These hormones undergo crosstalk with various TFs and play a primary role in SE^[20]^. However, information on interactions between phytohormones and *WOX* genes in *P. bournei* is lacking. In our study, referring to the information of *cis-*acting elements in the promoters of *PbWOXs*, we evaluated that the expression profiles of *PbWOXs* in embryogenic calli after treatment with IAA, MeJA, and ABA.

Auxin was first discovered to affect embryonic initiation in carrot and has been widely used to induce SE not only in angiosperms but also in gymnosperms^[56]^. Moreover, studies have indicated that auxin distribution is positively correlated with the accumulation of *WUS*, *WOX2*, and *WOX9* transcripts^[27,57,58]^. In *P. bournei*, the expression of *WUS* and *WOX5/7* was induced by auxin. However, the expression of *WOX2* and *WOX9* was inhibited, opposite to what has been reported in *Picea abies*^[49]^. In view of this phenomenon, we analyzed the possible causes of species differences or differences in auxin concentration. Whether* WOX2/WOX9* and auxin play a synergistic or antagonistic role in somatic embryo initiation remains to be determined.

ABA involvement in embryo development and maturation has been demonstrated in the SE of several species. In late embryonic development, LEA proteins accumulate in large quantities and act as components of ABA-inducible systems. On the other hand, exogenous ABA in culture media has been shown to promote the maturation and regeneration of somatic embryos^[55]^. Previous studies have shown that embryo cells cultured in media supplemented with 100 μM ABA produced more embryos in sugi^[59]^. Six *SlWOXs* were significantly upregulated after 3 h of 100 μM ABA treatment in tomato^[60]^. Our data showed that *PbWOX11/12a*,* PbWOX11/12b* and *PbWOX11/12c* were also briefly induced after 3 h of ABA treatment. At the same time, these three genes were highly expressed in the cotyledon-producing embryo stage of SE. It was further speculated that the expression of *PbWOX11/12s* is likely to be activated by ABA signaling, thereby promoting somatic embryo maturation.

MeJA is another hormone that increases somatic embryo induction and maturation rate. The effect of MeJA is similar to that of ABA to some extent, but it cannot replace ABA^[33]^. Previously, 50 μM and 100 μM MeJA were used to treat embryonic calli of longan^[61]^, and exogenous applications of 10-400 μM MeJA produced more mature somatic embryos to different extents^[62]^ Here, we used qPCR to measure the expression changes of *PbWOXs* after 100 μM MeJA treatment and hoped to determine the relationship between *PbWOXs* and MeJA. Our data showed that the expression levels of *PbWUS* and *PbWOX11/12a* were induced rapidly after MeJA treatment. In addition, the expression levels of *PbWOX2a* and* PbWOX9* were inhibited by MeJA after 3 h. Therefore, applying MeJA to calli for a suitably short time might promote the somatic embryo differentiation process in *P. bournei*.

## CONCLUSIONS

The WOX family is unique to plants, and WOX members play important regulatory roles in plant development, such as embryonic patterning. In the present study, we identified 15 *PbWOX* members in* P. bournei*, and their expression patterns among different tissues and SE process were determined, and the relationships between *PbWOXs* and hormones were also analyzed. These results are helpful to further study the regulatory roles of *PbWOXs* during SE*,* thus provides the important gene resources for regulating the SE process in *P. bournei* and other forestry trees.

## SUPPLEMENTARY DATA

Supplementary data to this article can be found online.
