# Supplementary material for: Identification of WUSCHEL-related homeobox (WOX) gene family members and determination of their expression profiles during somatic embryogenesis in Phoebe bournei
Source: For Res (Fayettev). 2023 Feb 28;3:5. doi: 10.48130/FR-2023-0005 (PMC11524275; doi:10.48130/FR-2023-0005)
Supplement: Supplementary file 1 — Supplementary data to this article can be found online. [file FR-2023-0005-Suppl-Table1.docx]

**Supplemental Table 1.** ***PbWOX* primers used for semi-qPCR and qPCR.**

| **Primer name** | **Primer sequences** |
| --- | --- |
| *PbWUS-F PbWUS-R* | TACTTGCTGTTGGGCAGATG  TTTCTGCTGCTGTGTTCGTC |
| *PbWOX1a-F PbWOX1a-R* | GGTAGCAGCGGTTTACAGAC  CCAAACTCTCAGCATCTCGT |
| *PbWOX1b-F PbWOX1b-R* | ACTCATGGCAGCGATTTCTT  TCTTGCACCCTTCCACTTGA |
| *PbWOX2a-F PbWOX2a-R* | CACCAATGTTGTTCGTGGA  ATGCCTGTTGGGTGTAGAGG |
| *PbWOX2b-F PbWOX2b-R* | TCCAAAATCAGATGCCAACA  CACAGCCTTCTGCCCATATT |
| *PbWOX3-F PbWOX3-R* | TCAACTAGATGGTGCCCCAC  AGAGTGTCCGATTTGGAAGC |
| *PbWOX4-F PbWOX4-R* | ACTGAATCCCCATCACCCAG  AAATATCCATGGCCTGCGTG |
| *PbWOX5/7-F PbWOX5/7-R* | GGTGCAAATGTGGGAGAGTG  CCTCCTCCTCCGTAGCTATT |
| *PbWOX9-F PbWOX9-R* | TATGTTGCTGCCACTTCTGC  GACAGCCTCTTCTCCAAACG |
| *PbWOX11/12a-F PbWOX11/12a-R* | GAGCAAGTGAGGTCTAGGTG  ATTGGCATCTGCTACTGAGC |
| *PbWOX11/12b-F PbWOX11/12b-R* | GCTAGCCTCTCCATGAGTGC  CAACTCTGGCAAACCCATTT |
| *PbWOX11/12c-F PbWOX11/12c-R* | GCTAGCCTCTCCATGAGTGC  ACCCATTTGCTGAGAGATCG |
| *PbWOX13a-F PbWOX13a-R* | GGCTGGGAAACGTGTACTGT  TTGGCCATGTTGTGTCAGTT |
| *PbWOX13b-F PbWOX13b-R* | TCGAAAGCAAATCTCCGTCT  TGCCTTGCGTTGATCTTATG |
| *PbEF1α-F PbEF1α-R* | CATTCAAGTATGCGTGGGT  ACGGTGACCAGGAGCA |
